# Association between germ-line HLA and immune-related adverse events

**DOI:** 10.3389/fimmu.2022.952099

**Published:** 2022-09-13

**Authors:** Ning Jiang, Yue Yu, Min Zhang, Yu Tang, Dawei Wu, Shuhang Wang, Yuan Fang, Yu Zhang, Lin Meng, Yingying Li, Huilei Miao, Peiwen Ma, Huiyao Huang, Ning Li

**Affiliations:** ^1^ Clinical Cancer Center, National Cancer Center/National Clinical Research Center for Cancer/Cancer Hospital, Chinese Academy of Medical Sciences and Peking Union Medical College, Beijing, China; ^2^ Oncology Bussiness Department, Novogene Co., Ltd, Beijing, China; ^3^ Research and Development Department, Burning Rock Biotech, Guangzhou, China

**Keywords:** immune-related adverse events, immune checkpoint inhibitors, human leukocyte antigen, PD-1, genotype

## Abstract

**Background:**

In recent years, significant progress has been made in immune checkpoint inhibitors (ICIs). However, accompanied by remarkable efficacy, a growing number of immune-related adverse events (irAEs) also arose. The mechanism of irAEs remains unclear. Previous studies indicated a positive association between specific human leukocyte antigen (HLA) variants and irAEs. Therefore, we planned and initiated a large cohort study aiming to uncover the relationship between irAEs and divergent HLA types.

**Methods:**

We screened all patients who have been treated in the clinical research ward, Cancer Hospital of the Chinese Academy of Medical Sciences. All participants were diagnosed with malignant tumors with complete AE follow-up data in the original electronic medical records. Sequencing libraries were generated using a customized panel, and four-digit formatted HLA alleles were extracted for further analysis. Association analysis was performed between HLA variants and different irAEs. We introduced two external reference groups and a non-irAE control group within the study cohort to control the type I error. We also explored the relationship between the zygosity of HLA genes, the evolutionary divergence of HLA class I genotype (HED), and irAEs.

**Results:**

530 participants received at least two doses of ICIs. The median follow-up time was 10.3 months. 97% of patients received anti-PD-1/PD-L1 treatment. The occurrence of overall irAEs showed no significant difference between the HLA homozygous group and the HLA heterozygous group. We did not find any significant association between irAEs and HED. We found that some HLA types are associated with irAEs of different organs and detected a significant association between HLA-DRB3*01:01 and thrombocytopenia (OR 3.48 (1.19,9.42), *p* = 0.011), HLA-DPB1*04:02 and hypokalemia/hyponatremia (OR 3.44 (1.24,9.1), *p* = 0.009), leukopenia (OR 2.1 (0.92,4.8), *p* = 0.037), anemia (OR 2.33 (1.0,5.41), *p* = 0.026), HLA-A*26:01 and bilirubin elevation (OR 2.67 (0.92,8.31), *p* = 0.037).

**Conclusions:**

IrAEs in specific organs and tissues may be associated with certain HLA types, while HLA heterogeneity has no significant influence on the happening of irAEs. More research is needed to explore the role of germline genetic changes in the risk assessment of irAEs.

## Introduction

Immune checkpoint inhibitors (ICIs) have opened a new chapter for cancer treatment as survival rates significantly improved with its application (1–4). However, accompanied by remarkable efficacy, immune-related adverse events (irAEs) also arose. IrAEs, ranging from the unnoticeable rash to irreversible visceral damage, can occur at any time throughout the treatment. Statistics have shown that severe irAEs affect up to 20-30% of patients treated total ICIs therapy (5), may lead to treatment discontinuation and also bring about intolerable physical morbidity, heavy financial burden, and even high risk of death (6–9).

Some pilot studies have explored the possible factors that may influence the development of irAEs, but its specific mechanism remains unclear (10, 11). The recent perspective regards irAEs as a feature related to the host immune function (particularly autoimmunity) rather than a pure tumor-oriented factor. The human leukocyte antigen (HLA), the molecules responsible for presenting peptide ligands to T cell receptor (TCR), are highly polymorphic, so variations in the peptide-binding region may represent different levels of binding affinity and highlight the abilities of divergent antigens (12). Certain HLA variants have been viewed as genetic risk factors for autoimmune diseases, such as HLA-B27 and ankylosing spondylitis, HLA-DR15 and multiple sclerosis, HLA-A*02:01, HLA-DR4, DR3, DQ2, HLA-DQ8, DR4/DQ4 and DR9/DQ9 (in Asian population) and type I diabetes, HLA-DR4 and rheumatoid arthritis (12, 13). HLA was also well established to be associated with autoimmune thyroid diseases, such as Hashimoto’s thyroiditis and Grave’s disease (14–17).

Similar to its role in autoimmune diseases, HLA may also play a role in the development of irAEs (18–23). However, the panoramic view of HLA molecules, their potential correlation with whole types of irAEs, and how HLA may relate to irAEs subtypes derived from different organs and systems remain to be explored. Therefore, we initiated the largest cohort study in this area, aiming to uncover the relationship between irAEs and the germline genetic signature of HLA, including HLA variants, the zygosity of HLA genes, and the evolutionary divergence of the HLA class I genotype (HED). We also explored the possible mechanism by which HLA causes irAEs using bioinformatics methods.

## Methods

### Study cohort

We have collected and analyzed the clinical data of 626 patients who have been treated in the clinical research ward at the Cancer Hospital of the Chinese Academy of Medical Sciences. All participants included in this study were diagnosed with malignant tumors and have received at least two cycles of immune checkpoint inhibitors with complete security follow-up data in the original electronic medical records. Patients with congenital or acquired immunodeficiency or those who had received allogeneic hematopoietic stem cell transplantation or a blood transfusion within 7 days were excluded from this study.

Blood samples were collected before the first cycle of treatment. The clinical data were collected from the original electronic medical records. In addition, the time of AE, laboratory and image results, AE treatment, influence on ICI treatment, and outcome of all AEs were collected for irAE identification. Furthermore, irAEs were judged by oncologists with experience in more than three clinical trials. Finally, irAEs and laboratory abnormalities were graded according to National Cancer Institute Common Terminology Criteria for Adverse Events (version 5.0).

To reduce the type I error and test the external consistency of research results, we introduced two external reference cohorts, the Novegene cancer cohort, and the Han-MHC reference panel, whose subjects did not overlap with the study cohort. Briefly, the Novegene control cohort includes 154 cancer patients with multiple cancer types. The Han-MHC reference cohort, which is reported by Zhou et al, includes 10,689 healthy individuals of Han Chinese ancestry (24). Only HLA typing data was retrieved and used for the two control cohorts.

The study protocol has been approved by the medical ethics committee of Cancer Hospital of the Chinese Academy of Medical Sciences. In addition, all patients in this study provided written informed consent for their blood samples to be used in our translational research.

### Library construction and sequencing

A total amount of 1.0μg genomic DNA per sample was used as input material for the DNA sample preparation. Sequencing libraries were generated using a customized panel which targeted approximately 1200 cancer and HLA genes. Briefly, a hydrodynamic shearing system (Covaris, Massachusetts, USA) carried out fragmentation to generate 180-280bp fragments. The remaining overhangs were converted into blunt ends *via* exonuclease/polymerase activities, and enzymes were removed. After the adenylation of 3’ ends of DNA fragments, adapter oligonucleotides were ligated. DNA fragments with ligated adapter molecules on both ends were selectively enriched in a PCR reaction. Then, captured libraries were also enriched in a PCR reaction to add index tags to prepare for hybridization. Products were purified using AMPure XP system (Beckman Coulter, Beverly, USA) and quantified using the Agilent high sensitivity DNA assay on the Agilent Bioanalyzer 2100 system. Libraries were sequenced with standard 2x150-bp paired-end reads on the Illumina NovaSeq 6000 sequencer.

### HLA typing and association analysis

Fastp (0.12.2) was used to remove low-quality reads and reads containing sequencing adapters. Then, clean data were imported into HLA-HD (v 1.4.0), a direct typing tool that uses next-generation sequencing date to analyze the HLA allele type (25, 26). Four digit formatted HLA alleles were extracted from HLA-HD results for further analysis. To access the enriched HLA alleles for irAEs, we chose irAE negative samples, irAE negative, or mild irAE samples, and set the Novegene cancer cohort and Han-MHC database as control groups (24).

### HED calculation

HLA evolutionary divergence (HED) was calculated as described by Pierini and Lenz (27). Briefly, protein sequences of each HLA class I allele (HLA-A, HLA-B, and HLA-C) were obtained from the IMGT/HLA database. The sequences of the variable regions in antigen-binding domains were extracted according to the specific motif of HLA-I genes. Next, divergences between allele sequences were calculated using the Grantham distance metric, which measures the physicochemical properties of amino acids. A custom Perl script was used to calculate the HED value for each HLA-I allele, and the input files include a FASTA file with aligned HLA alleles and a specific amino acid distance matrix. The mean HED was calculated as the mean divergence at HLA-A, HLA-B, and HLA-C. In all, we studied the association of HED with the clinical traits of irAE and cancer types.

### HLA binding model

NetMHCIIpan (v4.0) was applied to predict the binding peptides for HLA class II molecule (HLA-DRB3) (28). Protein sequences were downloaded from the UniProt database for six human platelet antigens (HPAs) genes: CD109, GP1BA, GP1BB, GP9, ITGA2, ITGA2B, and ITGB3 (29). We performed the binding prediction analysis considering all possible 15mer peptides. The predicted binding affinity (defined in predicted IC50) was ranked based on a comparison with a large pool of random peptides. The %rank threshold of 5 was used to screen binding peptides, and strong and weak binders were distinguished by the %rank threshold of 1. 3D modeling of HLA-DRB3 (2Q6W) was downloaded from the Protein Data Bank database (30). HLA-peptide binding structures were remodeled using a CABS-dock system. Electrostatic potentials around the resulting 3D structures were generated using the Adaptive Poisson-Boltzmann Solver (APBS) plugin within PyMOL (v2.5.2) (31).

### Statistical analysis

The Fisher-exact test was used to perform association analysis between HLA and different irAEs. Furthermore, we introduced two external reference groups to control the type I error. In addition, false discovery rate (FDR) correction was performed with the resulting p-values when comparing the HLA allele frequency between patients with irAEs and the control groups. Only HLA alleles with *p* value < 0.05 for all comparisons were considered significant. All statistical analyses involving HLA genotypes, HLA alleles, and clinical characteristics were performed in R environment, version 4.0.4. All derivative figures were generated using the package ‘ggplot2’, and the Limma package was used to perform tissue-specific gene expression analysis.

## Results

### Overview of clinical characteristics and irAEs in the study cohort

**Figure 1:**
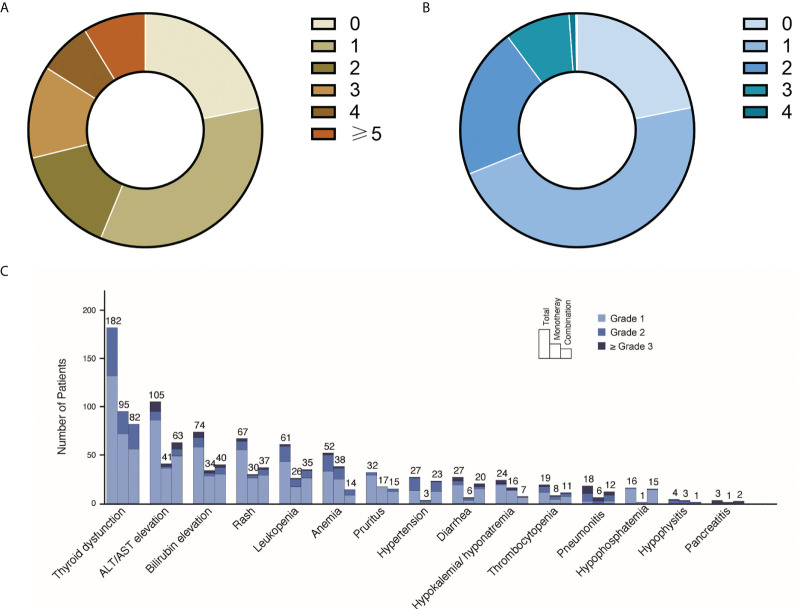
Landscape of irAEs distributions in the study cohort. **(A)** Number of irAEs in the study cohort. Among all 530 participants, 22% did not report any irAEs, 34% reported one irAE, 15% reported two irAEs, 13% reported three irAEs, 7% reported four irAEs, 9% reported ≥5 irAEs. **(B)** Highest grade of irAEs in the study cohort. 47% participants reported highest grade 1 irAEs, 21% reported highest grade 2 irAEs, 9% reported highest grade 3 irAEs and 1% reported highest grade 4 irAEs. **(C)** IrAEs in different organs/tissues. The numbers and grades irAEs of each organ/tissue are listed. For the PD-1/PD-L1 monotherapy and PD-1/PD-L1 and non IO combined therapy in this figure, all participants received PD-1/PD-L1 treatment (refer to **Table 1**).

Of the 626 patients, 530 were included in our study (refer to flowchart in **Supplementary Figure 1**). The median follow-up time was 10.3 (interquartile range 6.9, 21.7) months (refer to **Supplementary Figure 2** for details of the distribution of follow-up time). The demographics and clinical characteristics of the cohort are summarized in **Table 1**. Types of cancers included non-small cell lung cancer (NSCLC), small cell lung cancer (SCLC), esophageal cancer, gastric cancer, colorectal cancer, liver cancer, pancreatic cancer, biliary cancer, head and neck squamous cell carcinoma (HNSCC), nasopharyngeal cancer (NPC), breast cancer, renal cancer, bladder cancer, urothelial cancer, lymphoma, and cervical cancer. 97% of the patients received anti-PD-1/PD-L1 treatment. Of all participants, 78% reported irAEs of any grade, with 10.2% reporting grade 3 and above irAEs. Characteristics of irAEs in the study cohort are shown in **Figure 1**.

**Table 1:** Demographics and Clinical Characteristics of the study cohort (N=530).

	Number (%)
Median Age (interquartile range)	58 (50,65)
Sex	
Male	358 (68%)
Female	171 (32%)
Cancer type	
NSCLC and SCLC	129 (24%)
HNSCC and NPC	32 (6%)
Breast cancer	31 (6%)
Cancers of the digestive organs	260 (49%)
Cancers of the urinary system	14 (3%)
Lymphoma	18 (3%)
Cervical cancer	19 (4%)
Others	27 (5%)
TNM Stage	
I-III	79 (15%)
IV	449 (85%)
NA	2 (0.4%)
Previous Treatment	
0	159 (30%)
≥1 line	371 (70%)
Type of Current Treatment	
PD-1/PD-L1 monotherapy	248 (47%)
PD-1/PD-L1 and non IO combined therapy^a^	266 (50%)
PD-1/PD-L1 and additional IO combined therapy^b^	16 (3%)

a PD-1/PD-L1 combined with bevacizumab, TKIs and/or chemotherapies.

b Anti CTLA-4 and Anti LAG-3.

### Relationship between zygosity of HLA genes and irAEs

**Figure 2:**
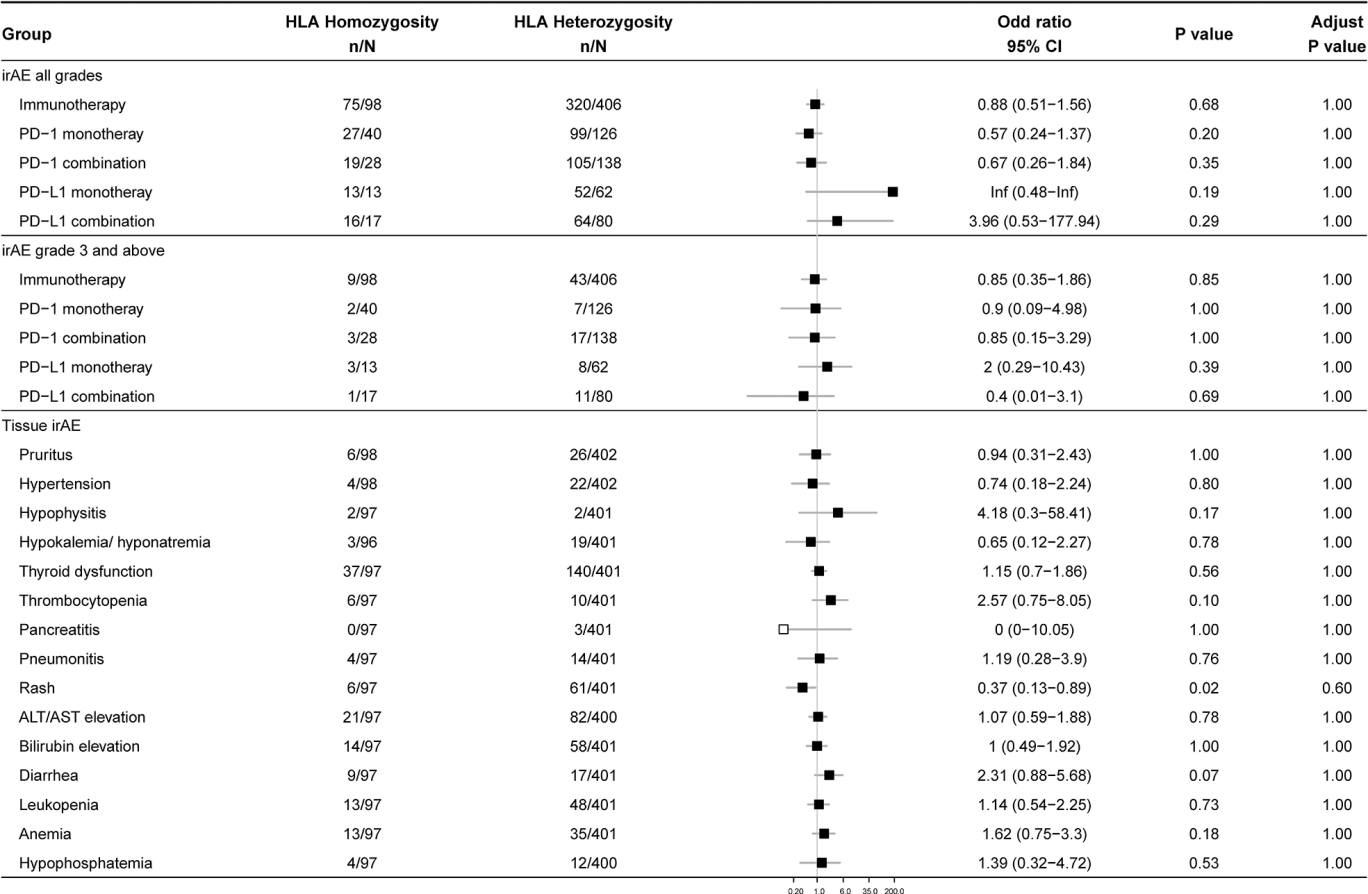
Association between zygosity at HLA genes and irAEs. Data show the occurrence of 1) overall irAEs, 2) grade 3 and above irAEs, and 3) irAEs of different organs and tissues in homozygous and heterozygous participants. The number of patients and odds ratio (OR) are highlighted. Horizontal lines represent the 95% confidence interval.

Since individual homozygous in HLA-I locus may present a smaller, less diverse repertoire of tumor-derived neoantigens, we hypothesized that heterozygotes in HLA genes might show a higher rate of irAEs. However, after the false discovery rate (FDR) correction, the overall occurrence of irAEs exhibited no significant difference between homozygous group and heterozygous group (**Figure 2**). Similar results were achieved in subgroups of monotherapy, combination therapy, irAEs with different grades, and irAEs in different organs and systems.

### Association between evolutionary divergence of HLA class I genotype (HED) and irAEs

**Figure 3:**
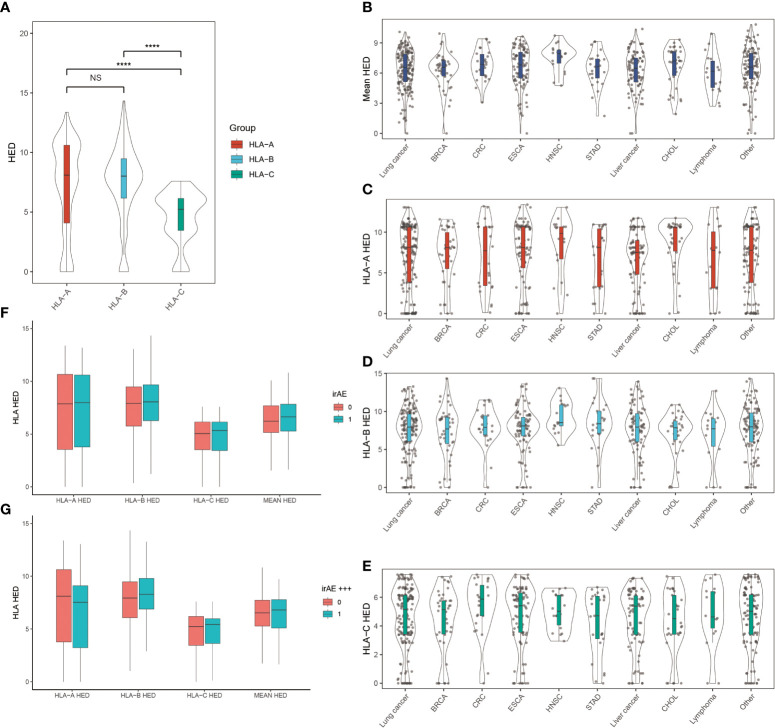
Association between HLA evolutionary divergence (HED) and irAEs **(A)** HED distributions among HLA-A, HLA-B, and HLA-C heterozygous genotypes. ****p< 0.0001. **(B)** Distribution of mean HED across different cancer types. **(C)** Distribution of HED at HLA-A across different cancer types. **(D)** Distribution of HED at HLA-B across different cancer types. **(E)** Distribution of HED at HLA-C across different cancer types. **(F)** Comparison of HED distributions between patients with and without irAEs. **(G)** Comparison of HED distributions between patients with and without ≥ grade 3 irAEs. NS, Not statistically significant (p ≥ 0.05).

Since higher sequence divergence between two alleles of HLA-I, namely the HLA-I evolutionary divergence (HED), may lead to more diverse antigenic peptide repertoire, we checked whether high HED is associated with more frequent occurrence of irAEs (**Figure 3**). We found that HLA-A and HLA-B showed higher HED than HLA-C in cancer patients. There is no significant difference in the HED of HLA-A, HLA-B, HLA-C, or mean HED among different cancer types. Contrary to our hypothesis, we found no difference in either mean HED or HED at HLA-A, HLA-B, and HLA-C. Neither did we find any significant differences in the distribution of HEDs when comparing patients with and without ≥ grade 3 irAEs.

### Relationship between HLA variants and irAEs

**Figure 4:**
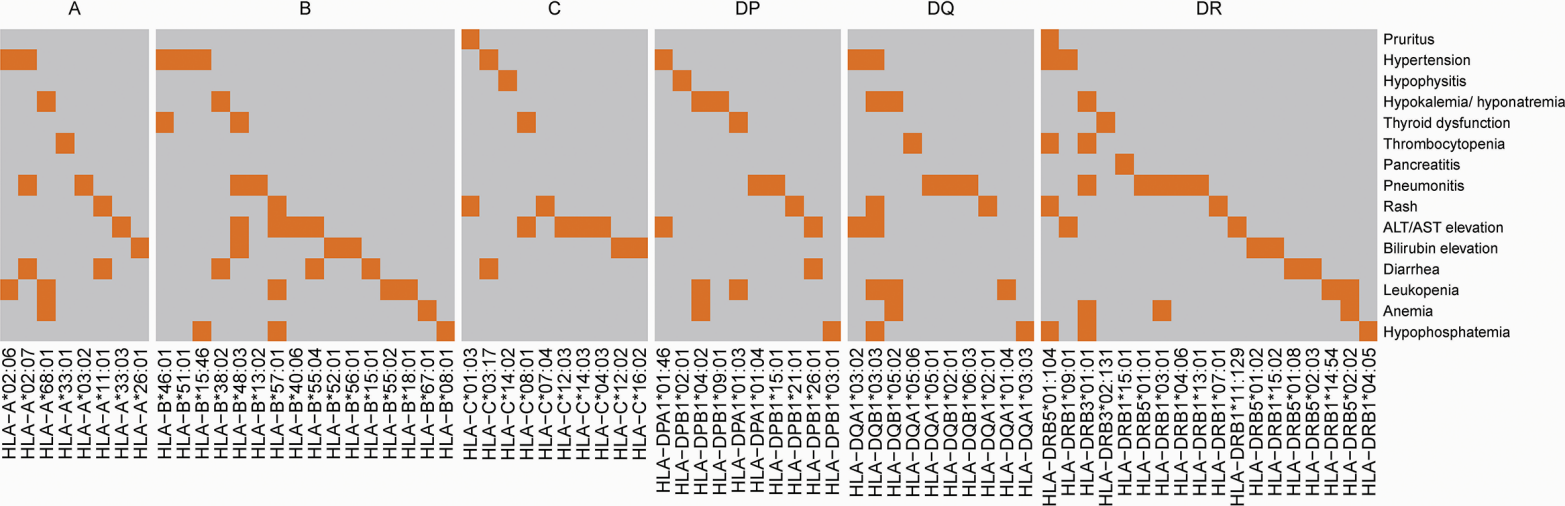
Association between HLA and irAEs in different organs. The highlighted area indicates that compared with subjects without AEs, patients with organ-specific AEs have a nominally significantly higher frequency of certain HLA types (p<0.05).

**Figure 5:**
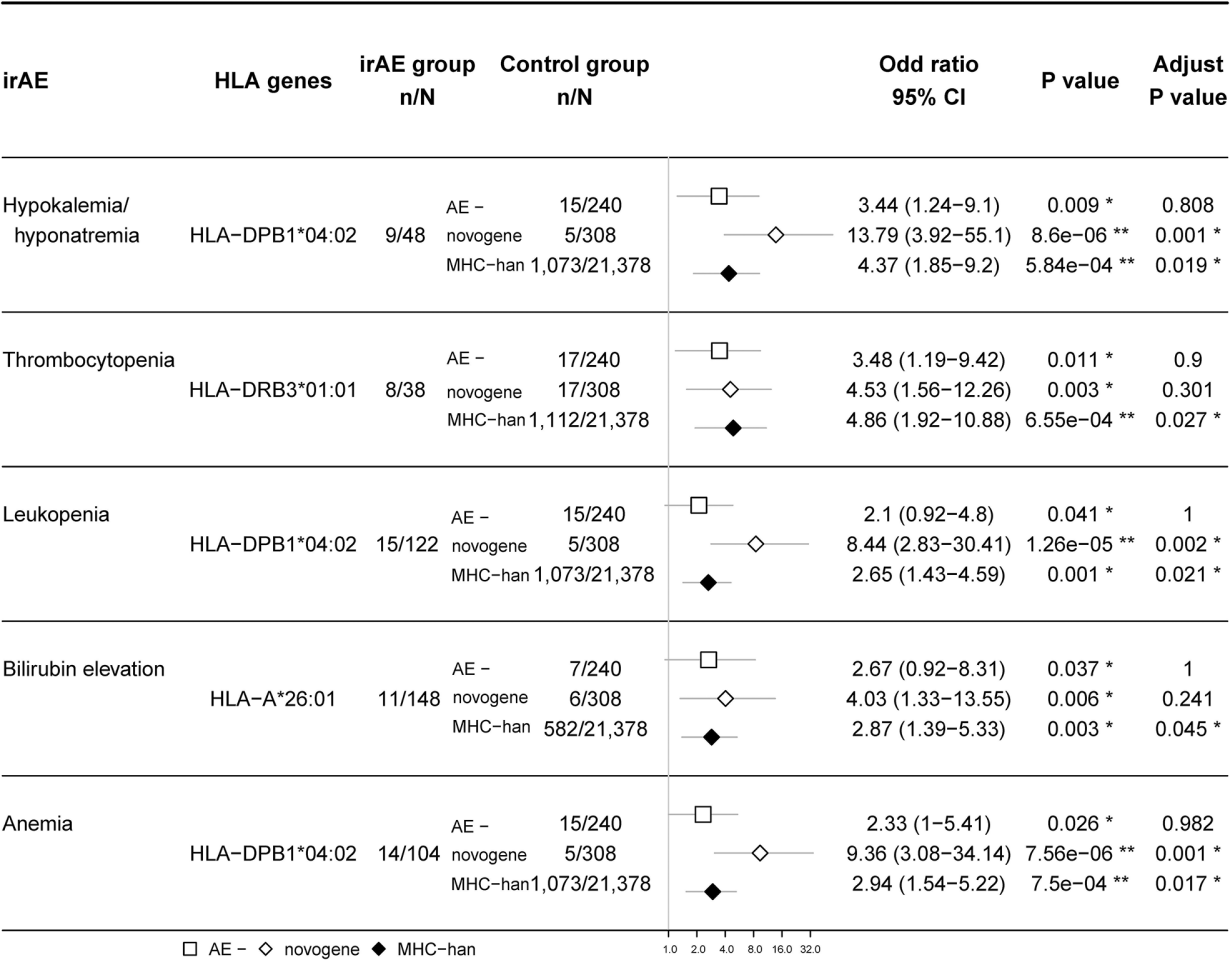
Association between certain HLA types and irAEs. The rates of suspected HLA types (gene frequency) in patients with irAEs are compared with 1) those in patients without any irAEs; 2) those in Novegene cancer cohort; 3) those in MHC-han Chinese reference cohort. False discovery rate (FDR) correction was performed with the resulting p-values when comparing the HLA allele frequency between patients with irAEs and the MHC-Han Chinese reference cohort. The number of patients and odds ratio (OR) are highlighted. Horizontal lines represent the 95% confidence interval.

Since a variety of autoimmune diseases are related to HLA typing, we hypothesized that irAEs and HLA typing are strongly associated. We first tested the association between HLA types and irAEs of different organs in our ICI treatment cohort (nominally significant HLA variants shown in **Figure 4**), then validated this association by comparing the frequency of HLA types between participants with irAEs and the Novegene cancer reference cohort and the healthy MHC-Han Chinese reference cohort. HLA variants were considered suspected when nominal *p* < 0.05 in comparison with all four control groups and FDR adjusted *p* < 0.05 compared to the MHC-Han reference cohort. We found several HLA types that are associated with irAEs of different organs (**Figure 5**), including significant associations between HLA-DRB3*01:01 and thrombocytopenia (OR 3.48 (1.19,9.42), *p* = 0.011), HLA-DPB1*04:02 and hypokalemia/hyponatremia (OR 3.44 (1.24,9.1), *p* = 0.009), leukopenia (OR 2.1 (0.92,4.8), *p* = 0.037), anemia (OR 2.33 (1.0,5.41), *p* = 0.026), HLA-A*26:01 and bilirubin elevation (OR 2.67 (0.92,8.31), *p* = 0.037). No association was revealed between overall irAEs and certain HLA variants.

Thyroid dysfunction is one of the most common irAE after PD-1/PD-L1 treatment. Our study found that some HLA subtypes were significantly associated with thyroid dysfunction, including HLA-B*46:01 (OR 2.34 (0.9,6.04), *p* = 0.042), HLA-C*08:01 (OR 2.02 (1.08,3.53), *p* = 0.014) and HLA-DPA*01:03 (OR 1.83 (1.13, 2.97), *p* = 0.007) (**Supplementary Figure 3**), though after FDR adjustment, these associations did not show statistical significance.

Furthermore, we identified that the existence of participants with short follow-up times might lead to more type II errors. To avoid inaccurate data, we conducted a subgroup analysis that only included participants with follow-up time > 90days (**Supplementary Figure 4**). The results of this sensitivity analysis confirmed that our main conclusions remained the same after excluding these individuals.

### Three-dimensional model of HLA binding to self-antigenic peptides

**Figure 6:**
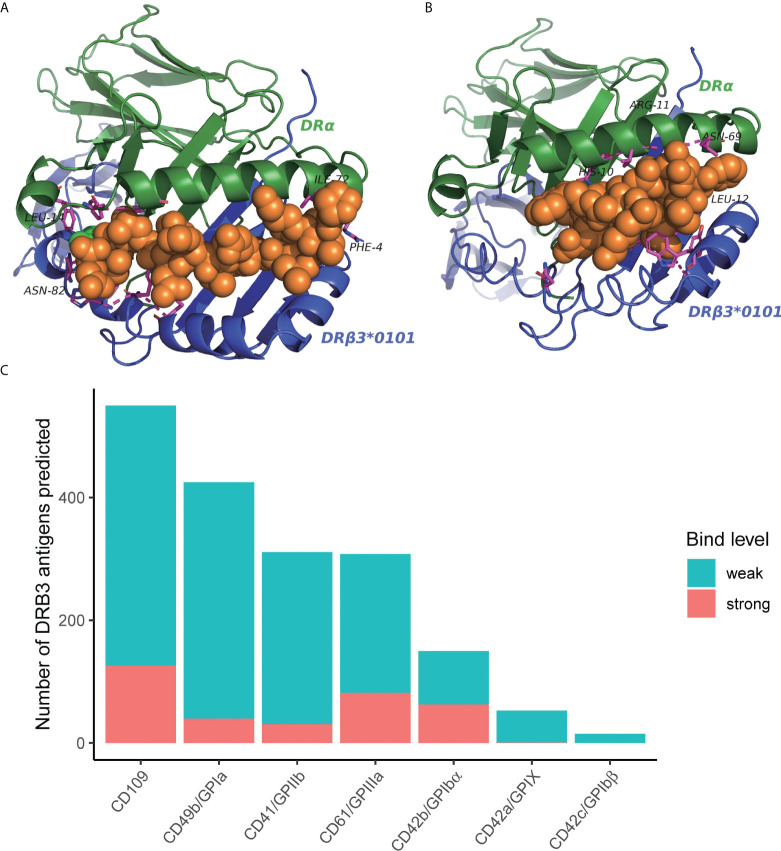
**(A, B)** Predicted three-dimensional model of HLA-DRB3*01:01 binding to possible self-peptides. HLA molecules, green and purple; bound self-antigenic peptides, yellow. **(A)** HLA-DRB3*01:01; PVKFLIDTHNRLLLQ (from CD109). **(B)** HLA-DRB3*01:01; KIGWRNDASHLL (from HPA-1a) **(C)** Number of binders to HLA-DRB3*01:01 from each human platelet antigen (HPA). The predicted binding affinity was ranked based on a comparison with a large pool of random peptides. The %rank threshold of 5 was used to screen binding peptides, and strong and weak binders were distinguished by the %rank of 1.

Considering the function of HLA in presenting antigen peptides, we hypothesized that the association between specific HLA variants and tissue-specific irAEs might be due to the ability to present self-antigens. To explore the possible HLA-self-peptide binding pattern, we tested the affinity between HLA and possible self-peptides using bioinformatics methods. Since antibodies against human platelet antigens (HPAs) are the cause of fetal and neonatal alloimmune thrombocytopenia, we virtually tested the affinity between suspected HLA molecules (HLA-DRB3*01:01) and all possible 15 aa peptides of six human platelet antigens (HPAs) using the NetMHCII (v4.0). We found multiple self-peptides that show a high predicted affinity (predicted IC50 <50nM) for suspected HLA types (**Supplementary Table 1**). Then, we further constructed three-dimensional models to show the binding pattern of HLA-DRB3*01:01 and self-peptides (**Figure 6**). HPA derived peptides bind to the pocket in the heterodimers complex. The prediction of binding motif was plotted as seglogo (**Supplementary Figure 5**).

## Discussion

In this study, we reported that some novel HLA variants may contribute to irAEs of various organs and systems, suggesting germline HLA types might be considered in irAE risk assessments.

In recent years, multiple studies have already unraveled the positive association between irAEs and HLA class I as well as HLA class II alleles, including HLA-DRB1*11:01 and pruritus, HLA-B27 and autoimmune encephalitis, HLA-DR15, HLA-Cw12 and adrenal insufficiency, HLA-Cw12, HLA-DR15, HLA-DQ7, and HLA-DPw9 with hypophysitis, HLA-DR4-DR53 and HLA-DR15 with thyroiditis, and HLA-B*35 and DRB1*11 with pneumonitis (18–21, 23, 32). The HLA- autoimmune disease association may vary according to different races/ethnicities. For instance, studies considering T1DM have shown that the distribution and effect size of disease-associated HLA types in Asian individuals might be different from those in Caucasian people (33–35). As the distribution and effect size of disease-associated HLA types might vary across different regions and populations (36, 37), we conducted the current study and found some novel suspected HLA variants, HLA-DRB3*01:01 associated with thrombocytopenia, HLA-DPB1*04:02 associated with hypokalemia/hyponatremia, anemia, leukopenia, and HLA-A*26:01 associated with bilirubin elevation.

In other studies, the HLA variants we found have also been associated with autoimmune diseases. For example, HLA-DRB3*01:01 is well established to be associated with fetomaternal alloimmune thrombocytopenia (38, 39). In addition, HLA-DRB3*01:01 is associated with type 1 autoimmune hepatitis and primary Sjögren’s syndrome (40). HLA-A*26:01 frequency was also increased in idiopathic hypoparathyroidism and Behçet’s disease (41–43).

We failed to find the association between HLA variants and the occurrence of overall irAEs. On the other hand, multiple HLA variants are associated with tissue and organ-specific irAEs. This result is consistent with the results presented by Ali et al. (18); they have previously reported no positive finding in terms of the full profile of irAEs (18). Since the process of molecular mimicry is tissue specific, HLAs that present certain self-peptides may only be involved in certain subtypes of irAEs. Heterogeneity of mechanisms of various irAEs might also be one of the reasons why we fail to identify HLA variants that are associated with overall irAEs. Loss of self-tolerance, molecular mimicry, epitope spread, inflammatory cytokine profile alteration, complement-mediated inflammation, and subclinical inflammation amplification may coexist, and the way these conditions impact different irAE subtypes may vary (10, 11, 44).

In this study, we also explored the possible pathogenic mechanism of HLA-DRB3*01:01 in thrombocytopenia using bioinformatics tools. Our results showed that HLA-DRB3*01:01 have a high predicted affinity with the self-peptides on human platelet antigens (HPAs), which may explain why a few patients suffered from thrombocytopenia after undergoing ICI treatment. Therefore, we raised the hypothesis that self-antigenic peptides may either be displayed by HLA-I molecules on the cell surface or presented by HLA-II positive APCs, which in turn activate autoreactive T cells. Still, *in vitro* and *in vivo* studies are still needed to verify the binding of autoreactive T cell-HLA-self antigen.

Since heterozygosity at classical HLA-I genes or high HED levels of HLA-B alleles had been found to be associated with improved survival in cancer patients treated with ICIs (45, 46), we assumed that individuals who are heterozygous for HLA or with high HED levels also have a higher incidence of irAEs. However, our outcomes pointed out that the correlation between heterozygosity and HLA certain types might be weak, regardless of treatment strategies and the irAEs subtype. Though epitope spread is one of the potential mechanisms of irAEs (44), the mechanisms responsible for irAEs are not identical to those for anticancer effects, and novel therapies might help manage irAEs without impairing the efficacy of antitumor treatment in the future.

In both the PD-1/PD-L1 monotherapy and combination therapy groups, approximately 5% of patients experienced new-onset hematological AEs of grade 1, and approximately 1% of patients experienced new-onset hematological AEs of grade 2 or higher, which was consistent with previous studies (47, 48). Multiple hematological diseases attributable to ICIs have been reported thus far, including aplastic anemia/pure red cell aplasia, autoimmune hemolytic anemia, autoimmune neutropenia, hemophagocytic lymphohistiocytosis, and immune thrombocytopenic purpura (47, 48).. However, the majority of patients with hematological AEs lacked bone marrow biopsy pathology results, though most anemia patients above grade 2 received the Coombs’ test, which was a limitation of our study as patients usually only had mild hematologic AEs. This was a limitation of our study. In this study, a significant correlation was found between HLA-DRB3*01:01 and thrombocytopenia, HLA-DPB1*04:02 and leukopenia, and anemia in ICI treatment. We believe that further research into the mechanisms underlying hematological irAEs could benefit from these associations.

According to a previous meta-analysis by Cantini L. and colleagues, using ICIs is associated with a greater risk of hypokalemia/hyponatremia compared to chemotherapy (49). Multiple potential mechanisms had been proposed (50). In our study, 3-4% of patients had hypokalemia/hyponatremia that were considered immune-related. In this study, we reported a significant association between hypokalemia/hyponatremia after ICIs use and HLA-DPB1*04:02. Additional research may reveal the novel mechanisms underlying hypokalemia and hyponatremia due to ICIs.

It is noteworthy that our study has limitations. Though this is the largest cohort exploring the association with HLA variants and irAEs up to now, considering the high polymorphism of HLA alleles and the possible association between rare irAEs and rare HLA variants, a larger multicenter cohort is needed to explore the landscape of the HLA-irAE link. We are now recruiting more participants to build a robust prediction model using HLA types and clinical characteristics. In this study, we found only four arthritis patients with positive RF or anti-CCP, and researchers did not initially consider mild seronegative arthralgia to be immune related in almost all cases. This led to arthritis being underestimated. The majority of patients with diarrhea at the cancer center did not undergo colonoscopy. Due to the above flaws in the execution of this study, we decided not to report the findings of genetic risk factors for these AEs. As the majority of irAEs lack specific markers, researchers may qualify non-irAEs caused by various factors as immune-related or irAEs as non-irAEs. This may result in an increase in false negative results and an underestimation of the odds ratio of certain genetic risk factors. In this study, we excluded patients who had only been treated for one cycle, so those who discontinued treatment at the initial stage because of irAEs could not be analyzed. All participants in the study cohort and two control cohorts were Chinese netizens, and further studies that may include different races/ethnic groups are needed to verify the suspected HLA subtypes reported by our team. However, despite this limitation, we believe that the results of the HLA heterogenicity/HED-irAE association might have a better external consistency.

In conclusion, our research sustains that irAEs in specific organs and tissues may be associated with certain HLA types, while HLA heterogeneity has no significant influence on the happening of irAEs. More studies are needed to explore the role of germline genetic changes in the risk assessment of irAEs, and potential therapies targeting irAE-inducing HLA types without influencing the anticancer may be considered to benefit patients.

## Data availability statement

According to national legislation/guidelines, specifically the Administrative Regulations of the People’s Republic of China on Human Genetic Resources (http://www.gov.cn/zhengce/content/2019-06/10/content_5398829.htm, http://english.www.gov.cn/policies/latest_releases/2019/06/10/content_281476708945462.htm), no additional raw data is available at this time. Data of this project can be accessed after an approval application to the China National Genebank (CNGB, https://db.cngb.org/cnsa/). Please refer to https://db.cngb.org/). Please refer to https://db.cngb.org/, or email: CNGBdb@cngb.org for detailed application guidance. The accession code HRA002676 should be included in the application.

## Ethics statement

The studies involving human participants were reviewed and approved by Medical ethics committee of Cancer Hospital of the Chinese Academy of Medical Sciences. The patients/participants provided their written informed consent to participate in this study.

## Author contributions

NJ, YY and MZ contribute equally to the study. NL: Conceptualization, Funding acquisition, Writing - Review & Editing. NJ: Methodology, Formal analysis, Data Curation, Investigation, Writing - Original Draft. YY: Investigation, Validation, Writing - Review & Editing, Resources. MZ: Methodology, Software, Formal analysis, Data Curation, Visualization. YT: Supervision, Project administration, Writing - Review & Editing. DW, SW, HM, PM, and YF: Resources, Investigation. YZ, LM, and YL: Software, Formal analysis, Visualization. HH: Writing - Review & Editing. All authors contributed to the article and approved the submitted version.

## Funding

This work was supported by Chinese Academy of Medical Sciences Innovation Fund for Medical Sciences (Platform Improvement of Clinical Trial Capability 2020-I2M-2-007); Chinese Academy of Medical Sciences Innovation Fund for Medical Sciences (Construction and application of clinical trial institution Evaluation System 2021-I2M-1-045); Beijing Municipal Science and Technology Commission (International Pharmaceutical Clinical Research and Development Platform 2015).

## Conflict of interest

Authors MZ, ML and YL are employed by Novogene Co., Ltd, Beijing, China. Author YZ is employed by Burning Rock Biotech, Guangzhou, China.

The remaining authors declare that the research was conducted in the absence of any commercial or financial relationships that could be construed as a potential conflict of interest.

## Publisher’s note

All claims expressed in this article are solely those of the authors and do not necessarily represent those of their affiliated organizations, or those of the publisher, the editors and the reviewers. Any product that may be evaluated in this article, or claim that may be made by its manufacturer, is not guaranteed or endorsed by the publisher.
