# Supplementary figures and images for: Association between germ-line HLA and immune-related adverse events
Source: Front Immunol. 2022 Sep 13;13:952099. doi: 10.3389/fimmu.2022.952099 (PMC9513190; doi:10.3389/fimmu.2022.952099)

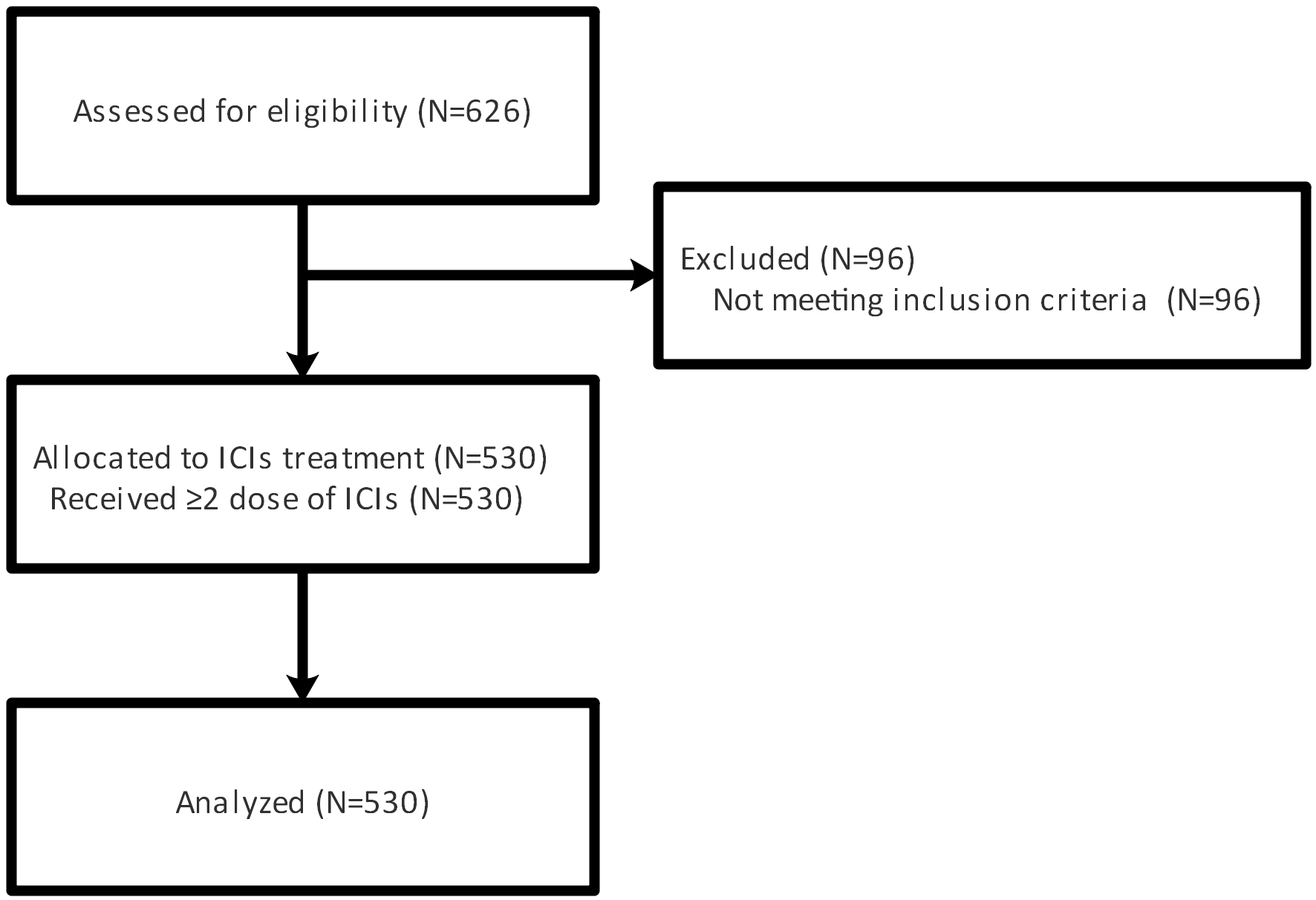

Supplement: Supplementary file 1 [file Image_1.tif]

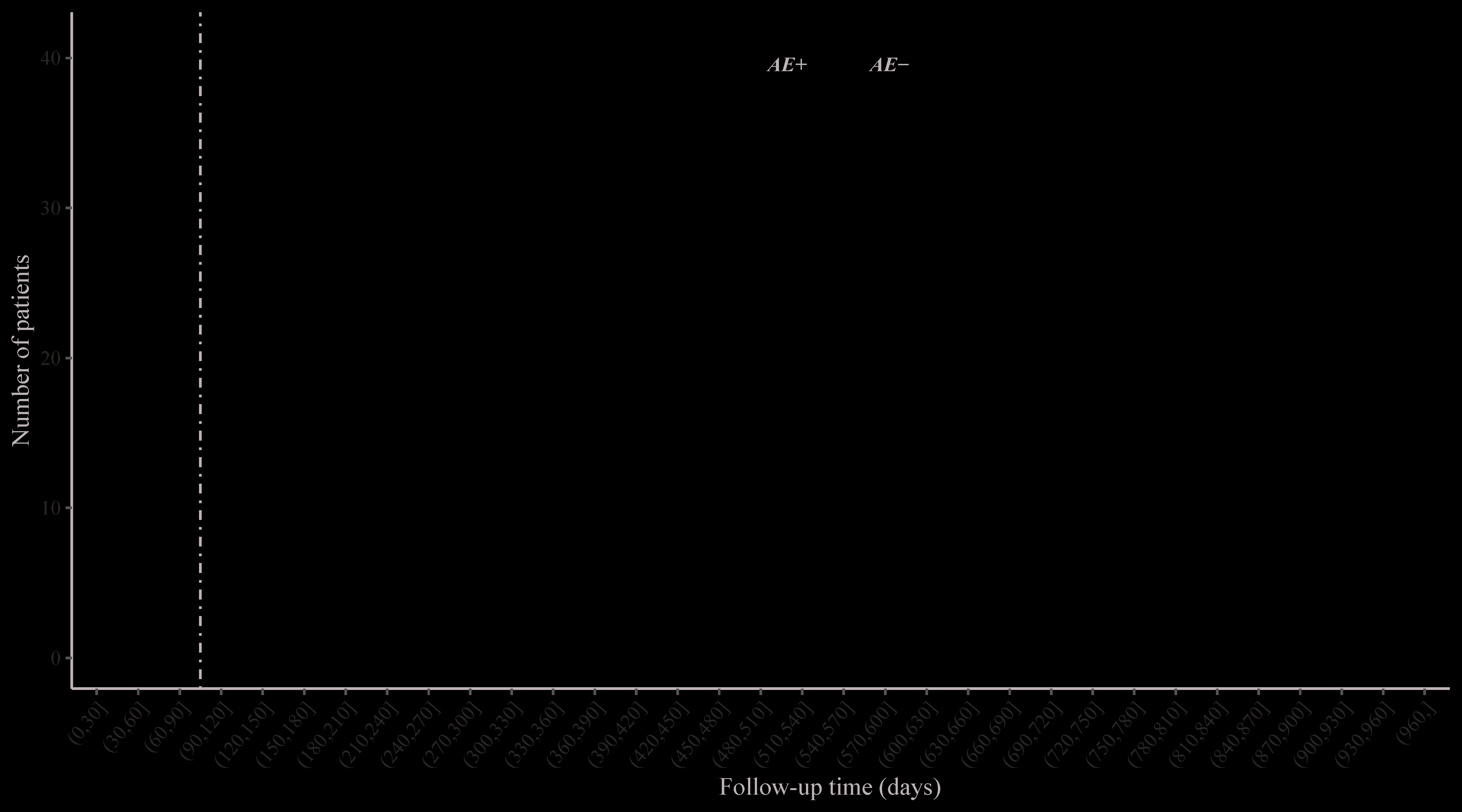

Supplement: Supplementary file 2 [file Image_2.tiff]

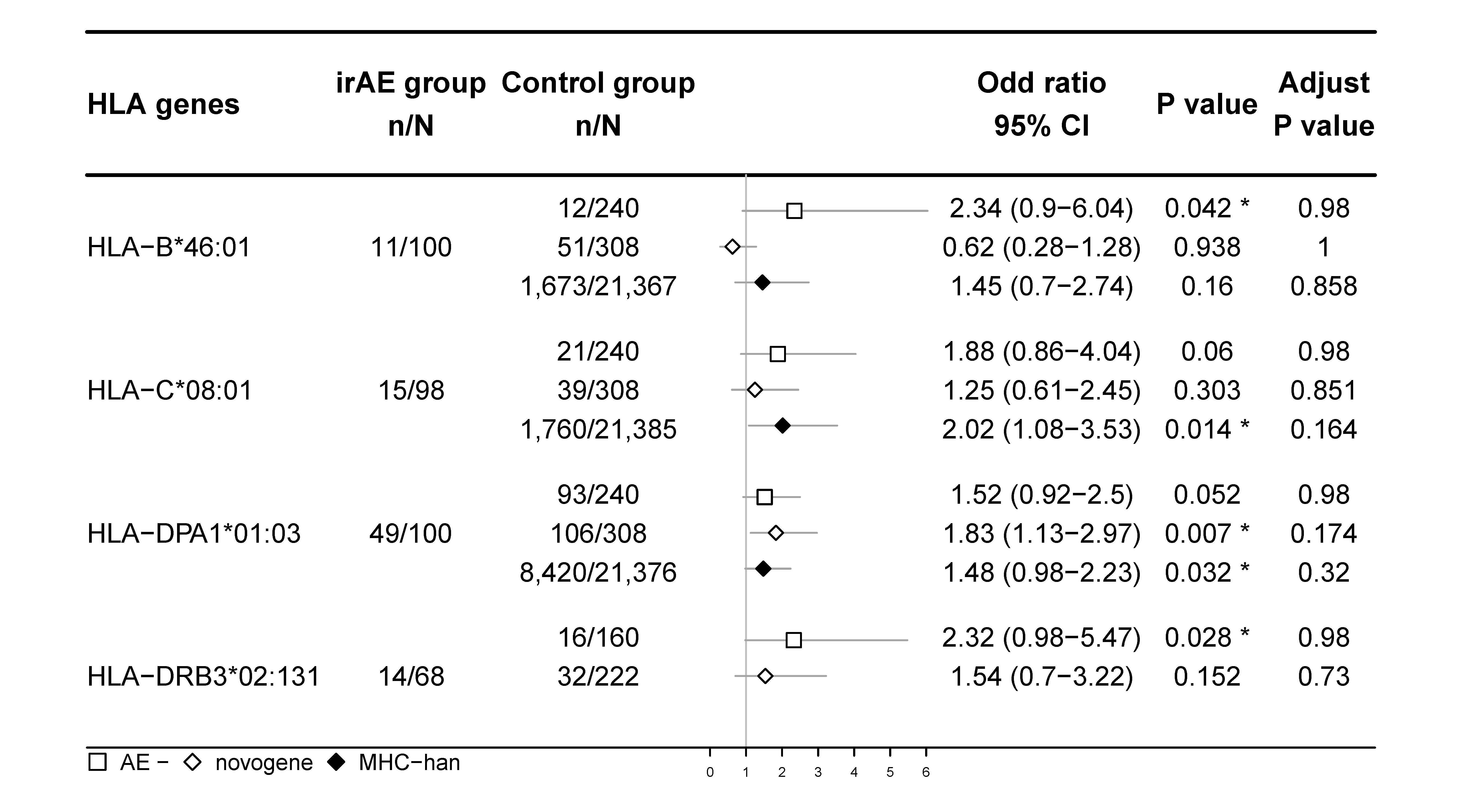

Supplement: Supplementary file 3 [file Image_3.tif]

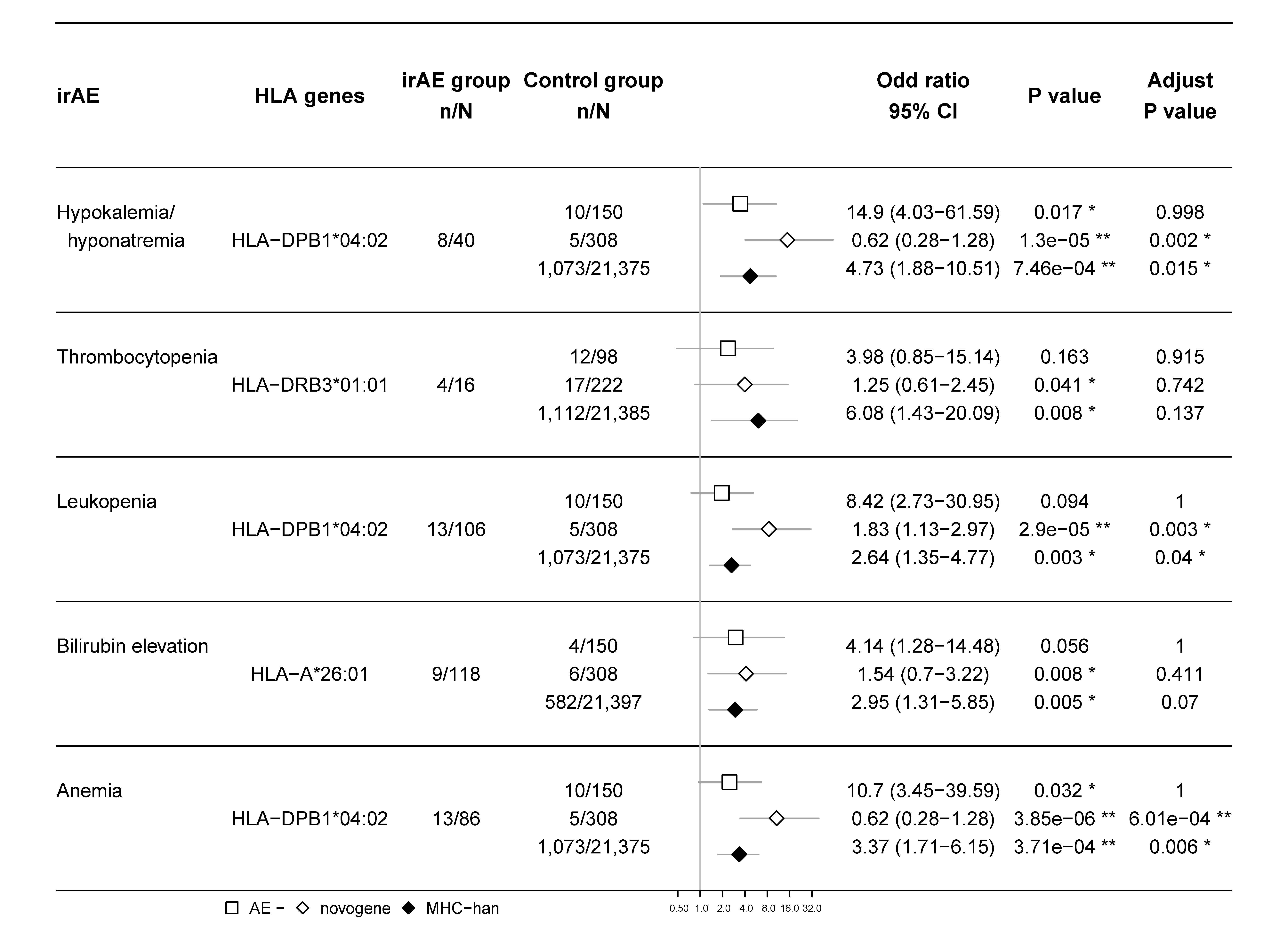

Supplement: Supplementary file 4 [file Image_4.tif]

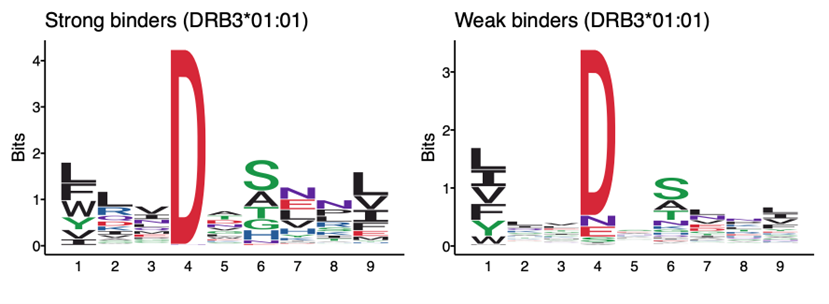

Supplement: Supplementary file 5 [file Image_5.tif]
